# Family History of Diabetes Is Associated With Delayed Fetal Postprandial Brain Activity

**DOI:** 10.3389/fendo.2018.00673

**Published:** 2018-11-20

**Authors:** Franziska Schleger, Katarzyna Linder, Laura Walter, Martin Heni, Johanna Brändle, Sara Brucker, Jan Pauluschke-Fröhlich, Magdalene Weiss, Hans-Ulrich Häring, Hubert Preissl, Andreas Fritsche

**Affiliations:** ^1^Institute for Diabetes Research and Metabolic Diseases of the Helmholtz Center Munich at the University of Tübingen, Tübingen, Germany; ^2^fMEG Center, Helmholtz Center Munich, University of Tübingen, Tübingen, Germany; ^3^German Center for Diabetes Research (DZD), Tübingen, Germany; ^4^Division of Endocrinology, Diabetology, Angiology, Nephrology and Clinical Chemistry, Department of Internal Medicine, University Hospital, Eberhard Karls University, Tübingen, Germany; ^5^Department of Obstetrics and Gynecology, University Hospital, Eberhard Karls University, Tübingen, Germany; ^6^Department of Pharmacy and Biochemistry, Faculty of Science, Eberhard Karls University, Tübingen, Germany; ^7^Institute for Diabetes and Obesity, Helmholtz Diabetes Center, Helmholtz Zentrum München, German Research Center for Environmental Health (GmbH), Neuherberg, Germany

**Keywords:** fetal MEG, oGTT, family history, type 2 diabetes, maternal metabolism, fetal programming

## Abstract

**Introduction:** We have previously shown that fetuses of mothers with gestational diabetes mellitus (GDM) and insulin resistance exhibit a prolongation of fetal auditory event-related brain responses (fAER) compared to fetuses of normal glucose tolerant women during an oral glucose tolerance test (oGTT). This implies that maternal metabolism may program the developing fetal brain. We now asked whether a family history of type 2 diabetes without metabolic programing also impacts fetal brain activity. We therefore investigated brain activity in fetuses of normal glucose tolerant mothers with and without family history of type 2 diabetes (FHD+ and FHD–).

**Methods:** A 75 g oGTT was performed in healthy pregnant women. Plasma glucose and insulin levels were measured after 0, 60, and 120 min. Each blood draw was preceded by fetal magnetoencephalographic (fMEG) recordings of fAER. From a group of 167 participants, a subsample of 52 metabolically healthy women, 37 with a negative, and 15 with a positive FHD (at least one first- or second-degree relative) was carefully selected based on the following inclusion criteria: inconspicuous pregnancy, no GDM, BMI 18.5–30 kg/m^2^, no preterm birth and at least two fMEG with detectable fetal responses during oGTT.

**Results:** An ANOVA showed a significant interaction between fMEG measurement time during the oGTT and FHD on fAER latency [*F*_(2)_ = 4.163, *p* = 0.018]. Fetuses of mothers with FHD+ had a prolonged fAER (273 ± 113 ms) compared to fetuses of mothers with FHD– (219 ± 69 ms) at 60 min during the oGTT [*F*_(1)_ = 4.902, *p* = 0.032]. There were no significant differences in age, BMI before pregnancy, weight gain during pregnancy and gestational age between the groups. Maternal glucose levels and insulin sensitivity were also not significantly different.

**Discussion:** In addition to the previously shown influence of maternal metabolism on fetal brain activity, maternal family history of diabetes (FHD) is also linked to fetal postprandial brain activity. This indicates that genetic and/or epigenetic factors modulate the postprandial brain response of the developing fetus.

## Introduction

A family history of type 2 diabetes (T2D) is associated with increased risk for diabetes in the offspring. This is assumed to be partially due to genetic factors and partially due to familial life style factors, including preconceptional lifestyle of parents (1, 2). In a population-based study (3) adult offspring with maternal or paternal diabetes had a 3-fold increased risk to develop T2D themselves, this risk doubled when both parents had diabetes. With an early onset maternal diabetes (before 50 years of age), the risk increased almost 10-fold. The authors attributed this further risk increase to perinatal exposure. The EGIR study (4) showed evidence for insulin resistance in a large group of healthy individuals without diabetes with a family history of T2D. Our group recently showed that a family history of diabetes (FHD) is also associated with a 40% increased risk for the prediabetic state (1). Finally, FHD had also been shown as an important risk factor for disturbed glucose metabolism during gestation (5).

There is evidence that the intrauterine environment influences the offspring's risk for obesity and T2D in later life. Maternal gestational diabetes mellitus (GDM) increases the risk for both diseases (6) independently of genetic or environmental background (7). We have previously shown that GDM of the mother influences brain responses of the human fetus. To study this, we used fetal magnetoencephalography (fMEG), which is a non-invasive technique to measure fetal brain activity (8). Fetal response latencies to auditory and visual stimuli measured with fMEG are altered in clinical conditions like intra-uterine-growth restriction (9, 10). This is also the case in fetuses of mothers with GDM in the postprandial condition after a 75 g glucose challenge in response to auditory stimulation (11). While in normal glucose tolerant mothers, fetal auditory event-related brain responses (fAER) show a decrease in latency from fasting to 60 min during an oral glucose tolerance test (oGTT) (12), latencies do not decrease in fetuses of mothers with GDM, and are longer than those of normal glucose tolerant mothers (11). Interestingly, this postprandial effect in the fetus is moderated by maternal insulin resistance (11, 12). We speculated that this postprandial effect reflects the regulatory ability of the fetal brain in regard to the peripheral metabolism. Possible explanations for the change in neuronal activation could be an effect of an increase of fetal glucose and/or insulin, or fetal insulin resistance induced by maternal hyperglycemia. Permanent hyperinsulinemia in the fetus might lead to peripheral and central desensitization. We hypothesize that postprandial AER latencies are an indicator for fetal central insulin resistance (11).

Having thus shown an effect of maternal metabolic parameters on fetal brain activity, we aimed to determine whether a FHD, without a phenotype of GDM in women, might impact the fetal brain response. We hypothesized that a FHD of the mother is associated with a fetal postprandial alteration of response latencies to auditory stimuli measured with fMEG similar to the one we see in women with GDM (11). We aimed to study the influence of FHD on the fetus, which might be via genetic or epigenetic influences, and therefore meticulously controlled for the influence of any maternal metabolic disturbances.

## Materials and methods

### Participants

We selected participants from our observational study on the influence of maternal glucose metabolism on fetal brain activity, where fetal brain activity is measured with fMEG and mothers are metabolically phenotyped: A 75 g oGTT was administered to healthy pregnant women with uncomplicated singleton pregnancies. Glucose and insulin levels were measured after 0, 60, and 120 min. Each blood draw was preceded by magnetoencephalographic recordings.

**Figure 1:**
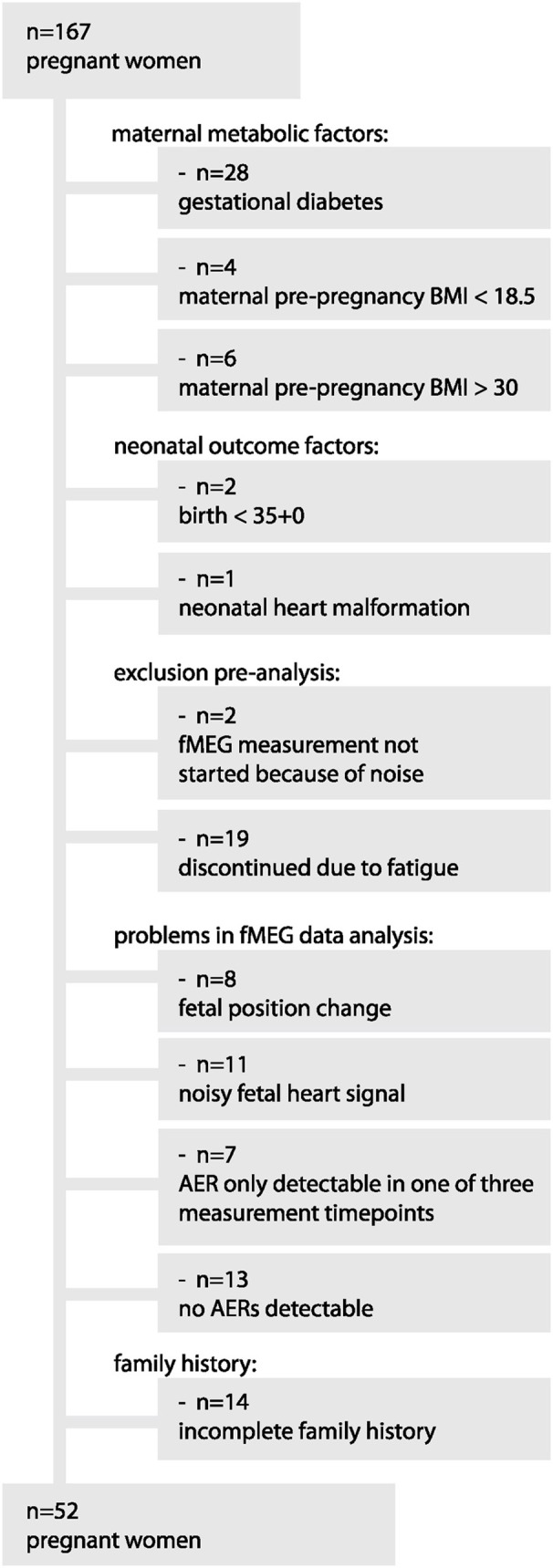
Exclusion process from original sample to final subsample.

From a group of 167 participants, a sub-sample of 52 women was selected based on the following inclusion criteria: maternal: no GDM, no pre-pregnancy BMI < 18.5 or > 30 kg/m^2^, fetal: at least two out of three fMEG recordings could be analyzed, neonatal: birth after 35 weeks of gestation, no serious clinical condition, and complete information for FHD for two generations (see Figure 1 for exclusion process).

The 52 women were categorized according to their family history, 37 with a negative (FHD–) and 15 with a positive family history of diabetes (FHD+). Of those participants with positive family history, 11 had one or more second degree relatives (grandparents), 4 had at least one first degree relative (father or mother or sibling). The mode of delivery of the 28 girls and 24 boys was: vaginal delivery *n* = 39, cesarean section *n* = 13.

Written informed consent of the participants was received prior to the measurements. The Ethical Committee of the Medical Faculty of the University of Tübingen approved the study plan.

### Paradigm

Measurements took place after an overnight fast of at least 5 h at the fMEG Center at the University of Tübingen. Women drank 75 g glucose solution (Accu-Chek Dextrose, Roche Diagnostics, Germany). Venous blood samples were obtained before ingestion of the glucose solution (0 min) and after 60 and 120 min. Each blood draw was preceded by an fMEG measurement. Fetal head position was determined via ultrasound before the first and after the last fMEG measurement.

### Laboratory measurements and calculations

Plasma glucose was determined in an ADVIA 1800 autoanalyzer (Siemens Healthcare Diagnostics, Erlangen, Germany) using the hexokinase method. Plasma insulin was analyzed using the ADVIA Centaur XP immunoassay system (Siemens AG, Erlangen, Germany). Plasma non-esterified fatty acids (NEFA) concentrations were measured enzymatically (WAKO Chemicals, Neuss, Germany) using the ADVIA 1800 analyzer (Siemens Healthcare Diagnostics, Eschborn, Germany). Maternal insulin sensitivity was calculated in units of μmol kg^−1^ min^−1^ pmol/l using the following formula: 0.156 – 0.0000459 × Ins_120_ – 0.000321 × Ins_0_ – 0.00541 × Gluc_120_ (12).

### fMEG measurements and analysis

fMEG data were recorded with the SARA system (SQUID Array for Reproductive Assessment, VSM MedTech Ltd., Port Coquitlam, Canada) as previously reported (11, 12). fAERs to a 500 Hz tone (duration 500 ms, intensity 95 dB, inter-stimulus-interval 1500 ms) were analyzed, based on the previously reported procedure (11, 12). The sound was generated by a speaker and transmitted by means of plastic tubing to an inflated plastic bag placed between the sensor array and maternal abdomen.

### Calculations and statistical analysis

Statistical tests were performed with SPSS (IBM SPSS Statistics for Windows, Version 20.0, Armonk, NY, United States). Results with *p* < 0.05 were regarded as statistically significant. Outcome measures were fAER latencies (ms), maternal blood glucose (mmol/l) and plasma insulin (pmol/l). Within-subject factor was measurement time during the oGTT and between subject factor was FHD group. Covariates were maternal age, maternal weight gain, maternal BMI before pregnancy and gestational age. For all 52 pregnant women, at least two of three fMEG measurements could be analyzed. At baseline, fAERs were not detectable in 13 fetuses, after 60 min in 8 fetuses and after 120 min in 5 fetuses. This detection rate is in the normal range for fetal MEG data (13). One insulin value was missing at 60 min. Missing values were replaced by series mean.

## Results

### Participants

The characteristics of 52 pregnant women and of the two subgroups with positive (FHD+) and negative family history of diabetes (FHD–) are shown in Tables 1, 2. The groups did not differ significantly in maternal age, maternal relative weight gain, gestational age, and birth weight.

**Table 1:** Descriptives of the *n* = 52 included participants.

	**Mean**	***SD***	**Minimum**	**Maximum**
Maternal age (years)	31	5	22	45
BMI before pregnancy	22.3	2.1	19.0	27.1
Gravidity	1.5	0.7	1	4
Parity	0.4	0.6	0	2
Birth weight (g)	3,368	490	2,150	4,450
Birth length (cm)	51	3	44	56
Gestational age (weeks)	30	2	28	36
**ORAL GLUCOSE TOLERANCE TEST**				
Blood glucose (mmol/l) 0 min	4.39	0.29	3.50	5.05
Blood glucose (mmol/l) 60 min	7.33	1.25	4.94	9.50
Blood glucose (mmol/l) 120 min	6.04	1.05	4.11	8.28

**Table 2:** Descriptives and comparison of the two subgroups with a negative family history of diabetes (FHD–, *n* = 37) and a positive family history of diabetes (FHD+, *n* = 15).

	**FHD–**		**FHD+**		p^*^
	**Mean**	***SD***	**Mean**	***SD***	
Maternal age (years)	32	5	30	5	0.242
BMI before pregnancy	22.3	2.1	22.4	2.2	0.855
Relative weight gain (kg/week)	0.32	0.09	0.26	0.12	0.075
Gravidity	1.6	0.7	1.4	0.6	0.342
Parity	0.4	0.6	0.3	0.5	0.395
Gender child (female/male)	22/15		6/9		0.202
Mode of delivery (vaginal delivery/cesarean section)	27/10		12/3		0.776
Birth weight (g)	3,413	492	3,275	484	0.303
Birth length (cm)	51	2	50	3	0.167
Gestational age (weeks)	30	2	30	2	0.687

* *t-test for normally distributed, Mann-Whitney-U for non-normally distributed variables, Pearson's χ^2^ to compare distributions*.

### Auditory measurements

There was no significant main effect of time (0, 60, 120 min) during oGTT on fAER latencies [*F*_(2)_ = 0.317, *p* = 0.729, repeated measures ANOVA], but a significant interaction between time and the presence or absence of FHD on fAER latencies [*F*_(2)_ = 4.163, *p* = 0.018]. This interaction remained significant when maternal age, maternal weight gain, maternal BMI before pregnancy and gestational age were included as covariates (*p* = 0.020).

**Figure 2:**
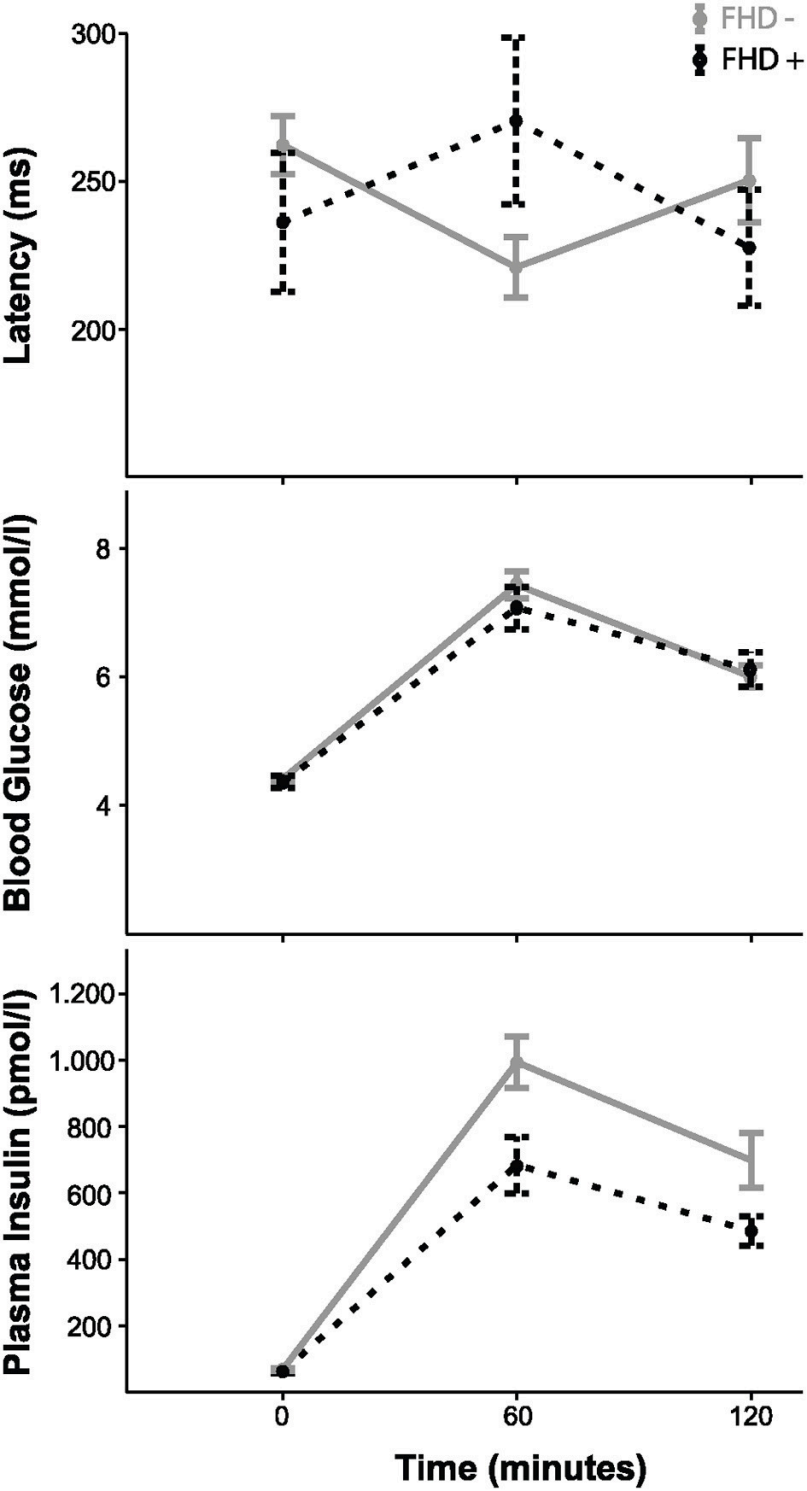
Fetal auditory event-related responses, maternal blood glucose and insulin over the course of the oGTT in the FHD– (*n* = 37) and FHD+ (*n* = 15) groups with mean ± 1 SEM.

*Post-hoc* tests showed that the glucose challenge leads to a postprandial change in latency in the FHD– but not in the FHD+ group [*F*_(2)_ = 4.624, *p* = 0.013; *F*_(2)_ = 1.088, *p* = 0.351; main effect of time on latency in repeated measures ANOVAs]. Latencies differ between fetuses of mothers with and without FHD at 60 min during oGTT, with longer latencies in fetuses of women with a FHD [*F*_(1)_ = 4.902, *p* = 0.032, univariate ANOVA with gestational age as covariate, see Table 3 and Figure 2].

**Table 3:** Maternal blood glucose and plasma insulin values and fetal latencies of auditory event-related brain responses for the two subgroups with a negative family history of diabetes (FHD–, *n* = 37) and a positive family history of diabetes (FHD+, *n* = 15).

**Oral glucose challenge**	**FHD–**		**FHD+**	
	**Mean**	***SD***	**Mean**	***SD***
Blood glucose (mmol/l) 0 min	4.41	0.27	4.35	0.36
Blood glucose (mmol/l) 60 min	7.43	1.24	7.07	1.28
Blood glucose (mmol/l) 120 min	6.01	1.07	6.11	1.05
Plasma insulin (pmol/l) 0 min	68	27	63	24
Plasma insulin (pmol/l) 60 min	994	475	669	335
Plasma insulin (pmol/l) 120 min	698	504	485	168
Insulin sensitivity	0.070	0.033	0.081	0.015
Latency (ms) 0 min	269	73	235	94
Latency (ms) 60 min	219	69	273	113
Latency (ms) 120 min	251	90	225	82

### Oral glucose challenge

As expected, glucose and insulin levels increased during the oGTT in both groups (FHD+, FHD–). There was no difference in the course of glucose levels during the oGTT between mothers with and without FHD [repeated measures ANOVA, interaction between time and group: *F*_(2)_ = 0.803, *p* = 0.451]. Accordingly, there was no statistically significant difference in the course of insulin levels during the oGTT between mothers with and without FHD [*F*_(2)_ = 2.887, *p* = 0.060]. There was no significant difference between groups in maternal insulin sensitivity measured by insulin sensitivity index (*U* = 0.818, *p* = 0.413). For maternal blood glucose and plasma insulin values for the family history subgroups, see Table 3.

## Discussion

In previous studies we have shown that maternal postprandial glucose metabolism influences fetal postprandial brain activity and the fetal autonomic system (11, 12, 14). Fetuses of mothers with gestational diabetes as well as fetuses of mothers with gestational insulin resistance exhibited longer latencies to auditory stimuli in the postprandial state. This implies impaired postprandial fetal brain activity in fetuses of mothers with impaired glucose regulation during pregnancy.

In the present study we now tested the hypothesis that genetic or epigenetic factors that increase the risk for diabetes will influence fetal postprandial brain response, independent of the actual metabolic status of the mother. To test this, we took advantage of our cohort of metabolically well phenotyped women with measurements of fetal postprandial brain activity with fMEG. We carefully identified samples of metabolically healthy normal glucose tolerant women with and without a family history of T2D, which were similar in subject characteristics and especially did not differ in glucose metabolism.

The groups differed specifically in the presence or absence of family history of T2D. In the absence of a direct measure, this was used as proxy for genetic or epigenetic risk for T2D (15). We found that fetuses of mothers with a positive FHD (similarly to fetuses of mothers with GDM) exhibit longer latencies to auditory stimuli in the postprandial state (60 min after ingestion of 75 g glucose) indicating impaired fetal postprandial brain activity in those fetuses.

While there was a trend toward a difference in the course of insulin levels during the oGTT between mothers with and without FHD, *p* = 0.060), they did not differ in maternal insulin sensitivity. In addition, postprandial insulin levels are low in this pregnant study cohort, and insulin sensitivity is four times higher (0.08) and therefore well in the normal range compared to the previously reported levels (0.02) in women with GDM (11). We therefore suggest that insulin levels do not influence group differences in latency in this specific insulin-sensitive study population.

Another variable that shows a tendency to be smaller in the FHD+ group is maternal weight gain (kg/week, *p* = 0.075). Since the interaction between time and family history of T2D on fAER latencies remained significant after controlling for weight gain, we assume that it also does drive the difference in fAER.

Latencies of auditory event-related responses of the fetal brain have been discussed in the context of brain maturation (8, 10, 11), with possible indications for later development. Studies on cognitive function in offspring of mothers with gestational diabetes are so far conflicting (16). At present, there are no long-term follow-up studies in offspring of mothers with and without a positive FHD.

Of note, the difference of fetuses with and without FHD is only detectable postprandially, 60 min after ingestion of 75 g glucose by the mother. This is in line with findings in GDM and gestational insulin resistance, where differences in brain response were also only detectable in a postprandial state. In the fasting state, we found no differences of fAER latency between groups, as in previous studies, we would argue that this indicates that challenges are necessary to determine alterations in fetal regulation abilities, and puts into question a possible predictive value for impaired cognitive function in offspring by baseline measures.

In our previous study (11) we speculated that the postprandial changes in activity might point to a functional effect related to postprandial maternal and fetal insulin resistance, possibly reflecting the ability of the fetal brain to regulate peripheral metabolism, called developmental origin of health and disease (17). Prolonged auditory event-related response latencies might be an early marker of this process. However, in the present data, the maternal metabolic influences are controlled by study design. We argue that this effect is only visible when samples are well-controlled for the stronger metabolic influence that usually overlays this effect. The difference of latencies is smaller in the present analysis of mothers with and without FHD (25%) than in the study of mothers with and without GDM (44%) (11). Therefore, the effect of GDM seems to be stronger than that of family history, e.g., genetic and epigenetic mechanisms.

We previously hypothesized that postprandial fAER latency is a proxy of fetal brain insulin resistance. In adult humans, we measured brain insulin action during oGTT (18) and after intranasal insulin application using magnetoencephalography and functional magnetic resonance imaging (19, 20). We hypothesized that brain insulin resistance may be induced in the fetal human brain with consequences for the glucose metabolism of the offspring in later life. We have previously shown that diabetes and obesity risk genes are associated with brain insulin resistance (21–23). Therefore, it can be speculated that the observed influence of a positive family history for diabetes on fetal latency represents the influence of diabetes risk genes on brain insulin resistance of the fetus. Another possibility is that the positive family history affects brain functions of the fetus via epigenetic mechanisms mainly mediated by lifestyle and diet of both father and mother (2, 24), which have been shown to adversely influence longterm risk of offspring metabolic morbidity (2).

In summary, a positive FHD of the mother seems to affect fetal postprandial brain function. Further follow up studies with the offspring have to show whether this altered brain reaction pattern in fetuses has consequences on the adult phenotype and whether it is reversible or not.

## Author contributions

FS and KL contributed equally to this work: substantial contributions to study design, acquisition, analysis and interpretation of data and drafting the article. LW contributed to data acquisition, analysis, interpretation. MH contributed to interpretation of data. JB, SB, JP-F, and MW contributed to data acquisition. H-UH contributed to study conception. HP and AF contributed to conception, design, interpretation and drafting of the article. All authors contributed to manuscript revision, read and approved the submitted version.

### Conflict of interest statement

The authors declare that the research was conducted in the absence of any commercial or financial relationships that could be construed as a potential conflict of interest.

## Abbreviations

fAER: fetal auditory event-related brain responses
FHD: family history of type 2 diabetes
FHD+: with a FHD
FHD–: without a FHD
fMEG: fetal magnetoencephalography
GDM: gestational diabetes mellitus
T2D: type 2 diabetes.
